# *Plasmodium falciparum*–Mediated Induction of Human CD25^hi^Foxp3^hi^ CD4 T Cells Is Independent of Direct TCR Stimulation and Requires IL-2, IL-10 and TGFβ

**DOI:** 10.1371/journal.ppat.1000543

**Published:** 2009-08-14

**Authors:** Anja Scholzen, Diana Mittag, Stephen J. Rogerson, Brian M. Cooke, Magdalena Plebanski

**Affiliations:** 1 Department of Immunology, Monash University, Alfred Medical Research and Education Precinct, Melbourne, Australia; 2 Department of Medicine, Royal Melbourne Hospital, University of Melbourne, Parkville, Australia; 3 Department of Microbiology, Monash University, Clayton, Australia; Case Western Reserve University, United States of America

## Abstract

CD4^+^CD25^+^Foxp3^+^ regulatory T cells (Tregs) regulate disease-associated immunity and excessive inflammatory responses, and numbers of CD4^+^CD25^+^Foxp3^+^ Tregs are increased during malaria infection. The mechanisms governing their generation, however, remain to be elucidated. In this study we investigated the role of commonly accepted factors for Foxp3 induction, TCR stimulation and cytokines such as IL-2, TGFβ and IL-10, in the generation of human CD4^+^CD25^+^Foxp3^+^ T cells by the malaria parasite *Plasmodium falciparum*. Using a co-culture system of malaria-infected red blood cells (iRBCs) and peripheral blood mononuclear cells from healthy individuals, we found that two populations of Foxp3^hi^ and Foxp3^int^ CD4^+^CD25^hi^ T cells with a typical Treg phenotype (CTLA-4^+^, CD127^low^, CD39^+^, ICOS^+^, TNFRII^+^) were induced. Pro-inflammatory cytokine production was confined to the Foxp3^int^ subset (IFNγ, IL-4 and IL-17) and inversely correlated with high relative levels of Foxp3^hi^ cells, consistent with Foxp3^hi^ CD4 T cell–mediated inhibition of parasite-induced effector cytokine T cell responses. Both Foxp3^hi^ and Foxp3^int^ cells were derived primarily from proliferating CD4^+^CD25^−^ T cells with a further significant contribution from CD25^+^Foxp3^+^ natural Treg cells to the generation of the Foxp3^hi^ subset. Generation of Foxp3^hi^, but not Foxp3^int^, cells specifically required TGFβ1 and IL-10. Add-back experiments showed that monocytes expressing increased levels of co-stimulatory molecules were sufficient for iRBC-mediated induction of Foxp3 in CD4 T cells. Foxp3 induction was driven by IL-2 from CD4 T cells stimulated in an MHC class II–dependent manner. However, transwell separation experiments showed that direct contact of monocytes with the cells that acquire Foxp3 expression was not required. This novel TCR-independent and therefore antigen-non specific mechanism for by-stander CD4^+^CD25^hi^Foxp3^+^ cell induction is likely to reflect a process also occurring *in vivo* as a consequence of immune activation during malaria infection, and potentially a range of other infectious diseases.

## Author Summary

Infection with the malaria parasite *Plasmodium falciparum* affects 300–600 million people each year. Regulatory T cells (Tregs) expressing the transcription factor Foxp3, which drives genes involved in immunosuppression, are specialized immune cells that can inhibit both protective and harmful inflammatory responses during malaria. While Treg numbers are increased during malaria infection, little is known about how they are induced by the parasite. We addressed this question using an *in vitro* culture system to model the interaction of the malaria parasite with human immune cells. We found that the parasite induced soluble immune mediators, including the T cell growth-factor IL-2 and the regulatory proteins IL-10 and TGFβ, which drive the induction and expansion of Tregs. These Tregs expressed high levels of Foxp3 and suppressed the production of inflammation and protective immunity-driving mediators by concurrently induced effector T cells. Importantly, we demonstrate that induction of Tregs by the malaria parasite did not necessarily require direct contact with antigen-presenting cells. Our findings suggest that the parasite induces Tregs in an antigen non-specific manner, which may explain why malarial immunosuppression is not confined to malaria-specific immune responses, and provide new insights into the mechanisms governing Treg induction during malaria infection, and potentially other infectious diseases.

## Introduction

Malaria is one of the most important infectious diseases in humans, affecting 300–600 million people annually with more than a million fatal cases per year [1]. Infection with the *Plasmodium* parasite occurs hand in hand with a disturbance of immune homeostasis. While malaria is associated with severe immune-mediated pathology due to excessive inflammatory responses to the erythrocytic stage of the parasite [2], impaired T cell responses are also a hallmark of acute malaria infection [3]–[7]. CD4^+^CD25^+^Foxp3^+^ regulatory T cells (Tregs) are important players in maintaining immune homeostasis and controlling excessive immune responses [8], and disproportionate induction or expansion of Tregs may also explain the immunosuppressive state during acute malaria infection [9]. Indeed, while functions attributed to these cells differ greatly depending on the experimental model used, increased numbers of CD4^+^CD25^+^Foxp3^+^ T cells have been found in both human [10]–[13] and murine [14]–[18] malaria infection. Increased levels of CD4^+^CD25^+^Foxp3^+^ T cells can result either from the expansion of thymic-derived natural Tregs or from the *de novo* induction from previously naïve T cells. The *de novo* induction of the Treg-associated transcription factor Foxp3 in naïve human CD4 T cells has to date mainly been examined in *in vitro* systems using artificial stimuli such as TCR ligation via anti-CD3 in the presence of exogenously added recombinant cytokines including IL-2 and TGFβ [19],[20]. The mechanisms of Treg induction by the malaria parasite, however, and the potential contribution of the above factors (TCR stimulation, IL-2 and TGFβ) remain unknown.

TGFβ as well as IL-10 are functional mediators of immunosuppression [20]–[25] and have additionally been shown to play an important role in controlling excessive immune responses during malaria infection [15], [26]–[32]. Moreover, production of IL-10 during the early acute phase of infection is associated with an increased proportion of splenic CD4^+^CD25^+^ T cells in non-lethal *P. yoelii* infection [28]. In humans, an early peak of bioactive serum TGFβ precedes the up-regulation of CD4^+^CD25^+^ T cells and Foxp3 mRNA levels in whole peripheral blood mononuclear cells (PBMCs) [11]. While these studies suggest the involvement of IL-10 and TGFβ in CD4^+^CD25^+^ Treg induction during malaria, no causal connection has been proven to date. Studies on human subjects often only allow looking at Treg numbers and cytokine levels at limited time points after infection and more importantly do not allow any intervention. Therefore, *in vitro* assays reflecting *in vivo* processes as closely as possible are needed to examine the mechanisms involved in the malaria parasite-mediated generation of CD4^+^CD25^+^Foxp3^+^ human T cells.

In this study, we have examined the contribution of monocyte-mediated TCR stimulation and cytokines including IL-2, IL-10 and TGFβ to the induction of CD4^+^CD25^+^Foxp3^+^ T cells using an *in vitro* co-culture system of trophozoite-stage malaria-infected red blood cells (iRBC) with PBMCs from healthy donors in the absence of any further stimulation or addition of exogenous cytokines. We found that iRBCs, at concentrations typically found during mild/uncomplicated human malaria infection [12],[13], dose-dependently induce two populations of CD4^+^CD25^hi^ T cells, which express Foxp3 at high or intermediate levels and have distinct cell-contact and cytokine requirements for their induction. High ratios of Foxp3^hi^∶Foxp3^int^ cells inversely correlated with the magnitude of effector cytokine production by Foxp3^int^ cells, consistent with suppressive activity of parasite-induced Foxp3^hi^ cells on concomitantly induced effector Foxp3^int^ cells. The specific dependence of iRBC-induced CD4^+^CD25^hi^Foxp3^hi^ T cells on IL-10 and TGFβ demonstrated here further highlights the importance of these immuno regulatory cytokines during malaria infection. Importantly, direct monocyte contact was only required with bystander CD4^+^ T cells for the generation of soluble mediators, but not with the future Foxp3 expressing T cells themselves. These findings have implications beyond malaria infection, as they demonstrate that Foxp3 induction, contrary to current belief, is not necessarily confined to cells subjected to direct antigen-presenting cell (APC) mediated TCR stimulation.

## Results

### Time- and concentration-dependent induction of CD25^hi^Foxp3^hi^ and Foxp3^int^ CD4 T cells by malaria-iRBCs

**Figure 1:**
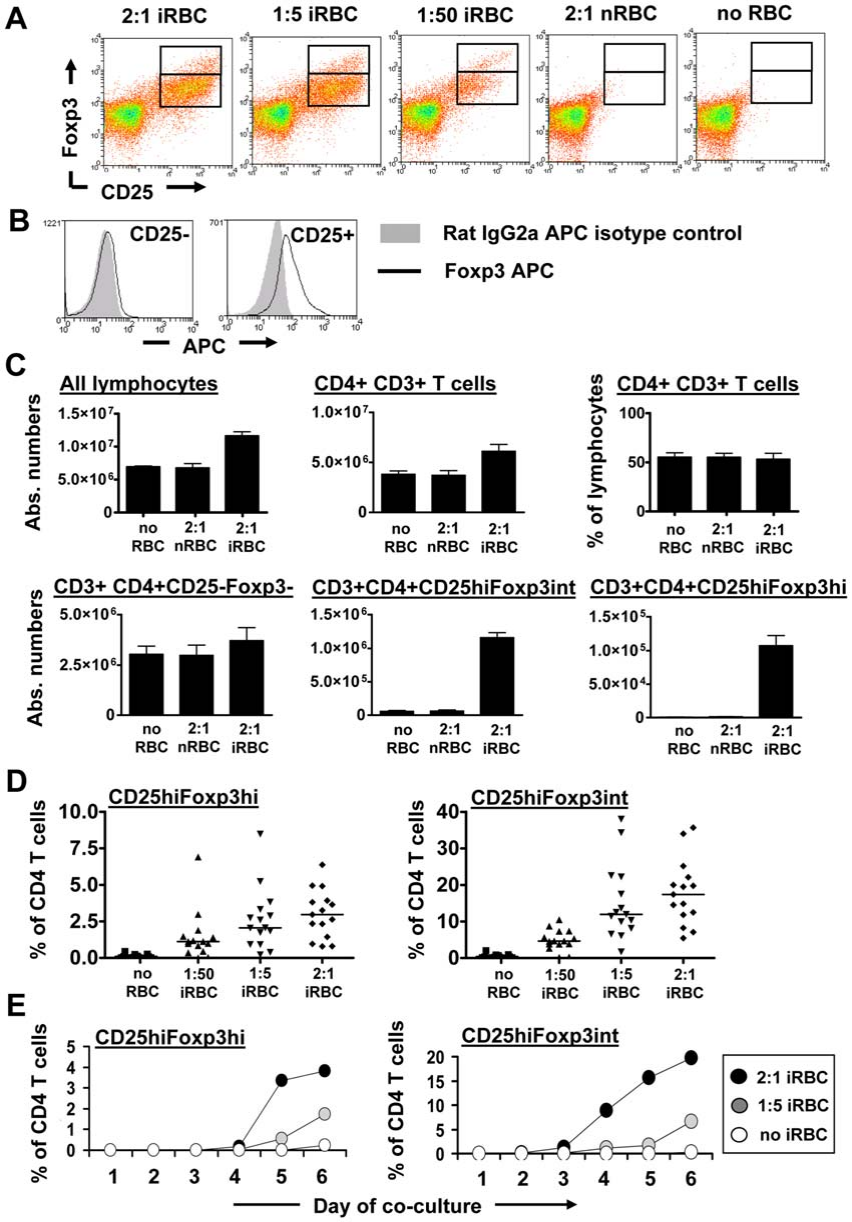
Induction of Foxp3^hi^ and Foxp3^int^ CD4^+^CD25^hi^ T cells by malaria-infected RBCs is time and concentration-dependent. PBMC were cultured alone, with uninfected RBCs (nRBC) or iRBCs for 6 days at different iRBC∶PBMC ratios. Cells were analysed by flow cytometry for CD25 and Foxp3 expression in gated CD4^+^CD3^+^ lymphocytes. Dot plots show 1 representative of 12 donors examined (A). Comparative analysis of Foxp3 and isotype control staining on CD4^+^CD25^−^ T cells and CD4^+^CD25^+^ T cells in day 6 cultures revealed specific Foxp3 expression solely in the CD25^+^ population (B). Absolute numbers of different CD4 T cell populations for 4 donors (Mean+/−SEM) were calculated based on light microscopic cell counts and flow cytometry analysis on day 6 of culture of originally 2×10^7^ PBMCs co-cultured in the absence or presence of nRBCs and iRBCs (C). Data from 12 donors demonstrate iRBC concentration-dependent induction of Foxp3^hi^ and Foxp3^int^ CD4^+^CD25^hi^ T cells (D). A representative plot of 1 out of 7 donors shows the induction of these cells over a time course of 6 days (E).

We first asked whether Foxp3 expressing CD4 T cells could be induced by culturing PBMCs of healthy Australian blood bank donors with malaria-infected trophozoite stage RBCs (iRBCs). Indeed, we observed an enhanced proportion of CD25^hi^Foxp3^+^ CD4 T cells, when PBMC were cultured in the presence of iRBCs but not normal RBCs (nRBC) for 6 days (Figure 1A). Within the CD25^hi^ gate, two populations could be distinguished based on their Foxp3 expression levels (Figure 1A). The existence of the two populations was further confirmed with a second anti-Foxp3 mAb (clone 259D, data not shown). While isotype/background staining was elevated on all CD25^+^ CD4 T cells in day 6 cultures, specific Foxp3 staining was only observed in the CD25^+/hi^ population (Figure 1B). There was no significant difference in the proportion of CD4^+^ T cells and the absolute number of CD4^+^CD25^−^Foxp3^−^ T cells recovered from PBMC cultured without RBCs or with nRBCs compared to iRBCs (Figure 1C), showing that the increase in Foxp3 expressing cells in iRBC∶PBMC co-cultures was not due to a loss of the CD25^−^Foxp3^−^ population. The higher recovery in absolute numbers of CD4^+^ T cells from iRBC∶PBMC co-cultures was due to the appearance of the CD25^hi^Foxp3^int^ and CD25^hi^Foxp3^hi^ populations (Figure 1C). For all further analysis, CD25^hi^Foxp3^int^ and CD25^hi^Foxp3^hi^ cells were analysed as the percentage of CD4 T cells, unless stated otherwise.

**Figure 2:**
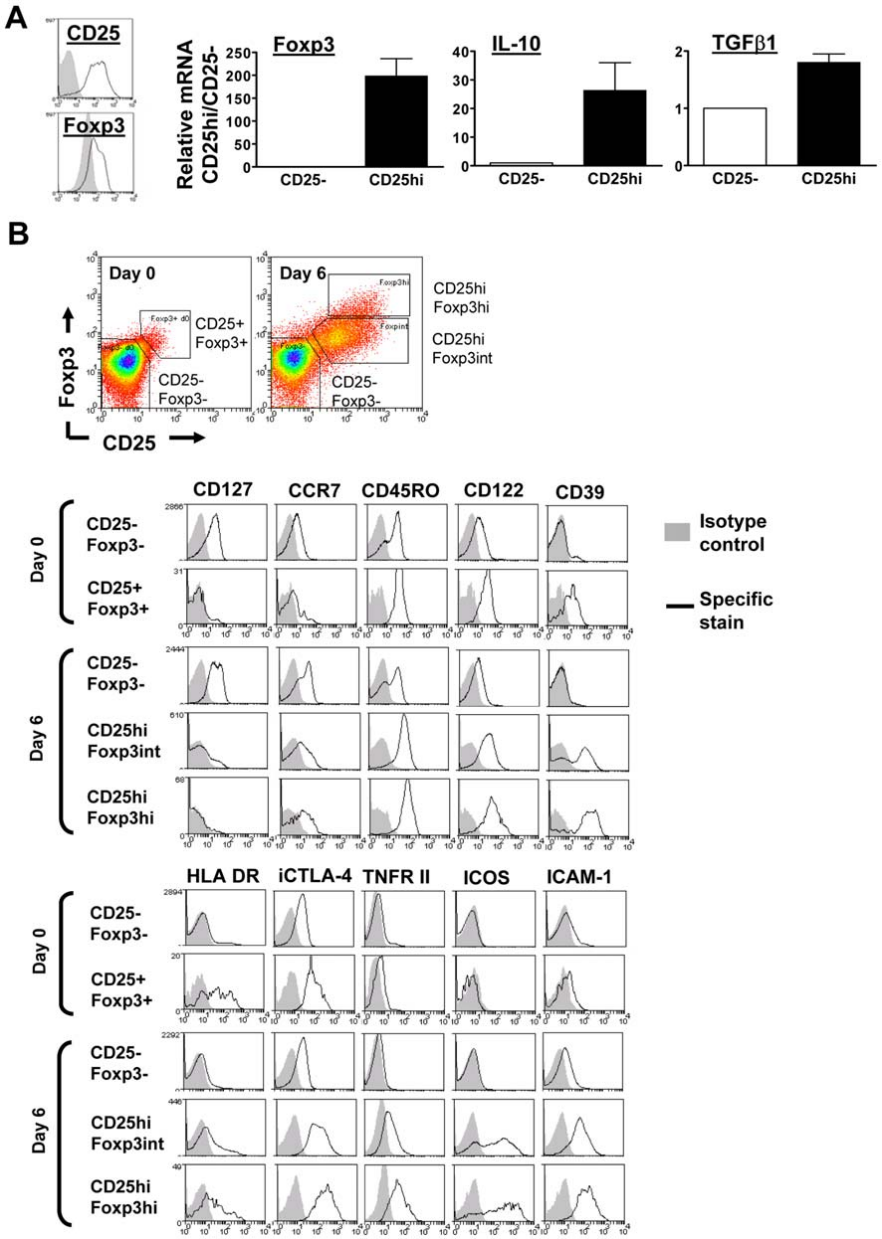
Phenotype of Foxp3^hi^ and Foxp3^int^ CD4^+^CD25^hi^ T cells induced by malaria-infected RBCs. After 8 days of iRBC∶PBMC co-culture, immuno magnetic selection was used to isolate CD4^+^CD25^hi^ (black line) and CD25^−^ cells (grey filled). CD25 and Foxp3 expression in the two sorted populations was analysed by flow cytometry (A). mRNA levels for Foxp3, TGFβ1 and IL-10 were normalized on 18SrRNA levels for each donor. Data from 8 donors (Mean+/−SEM) are shown, presented as relative mRNA levels normalized on mRNA levels in CD4^+^CD25^−^ T cells for each donor (A). PBMC of 3 donors were phenotyped on day 0 prior to iRBC-PBMC co-culture set up, and after 6 days of iRBC∶PBMC co-culture (2∶1 ratio). Dot plots show the gating strategies for Foxp3 expressing cells on day 0 and day 6, respectively (B). Representative histograms depicting isotype control staining (grey filled) and specific staining for various surface markers on CD25^−^Foxp3^−^ and CD25^hi^ Foxp3-expressing CD4 T cells (black line) from each respective culture are shown (B).

The percentage of induced CD25^hi^Foxp3^hi^ and CD25^hi^Foxp3^int^ CD4 T cells was dependent on the amount of iRBC added to the culture and increased with rising iRBC∶PBMC ratios (Figure 1D; *P*<0.0001, one-way ANOVA with linear trend post-test). The ratios used were calculated to reflect a range of natural concentrations typically found in the peripheral blood of malaria-infected individuals with low to medium parasitemia [12],[13] (0.001 to 0.1% parasitemia). Induction of CD25^hi^Foxp3^hi^ and CD25^hi^Foxp3^int^ CD4 T cells was detectable after 3–4 days of exposing PBMCs to iRBCs, and CD25^hi^Foxp3^int^ CD4 T cells were generally detectable 24 h earlier than the CD25^hi^Foxp3^hi^ population in 5 out of 7 donors examined (Figure 1E). Overall levels of Foxp3 expressing cells increased until day 6 of iRBC co-culture (Figure 1E), and were stable up to day 8 (3 out of 3 donors, data not shown). In co-cultures with nRBCs or in the absence of RBCs, CD25 and Foxp3 expression levels remained unchanged throughout the whole culture period (Figure 1E and data not shown) and were similar to those on freshly isolated CD4 T cells (Figure 2B). Unless otherwise stated, day 6 was used as the time point for analysis in subsequent experiments.

### iRBC-induced CD25^hi^Foxp3^hi^ and Foxp3^int^ CD4 T cells have a typical Treg phenotype

Specific Foxp3 expression in the induced CD25^hi^ CD4 T cells compared to CD4^+^CD25^−^ cells (*P* = 0.002) was further confirmed by quantitative real-time PCR analysis of MACS-sorted CD4^+^CD25^−^ and CD25^hi^ T cells. Additionally, iRBC-induced CD4^+^CD25^hi^Foxp3^+^ T cells also contained higher mRNA levels of the immunosuppressive cytokines IL-10 (*P* = 0.04) and TGFβ1 (*P* = 0.002) than CD25^−^Foxp3^−^ CD4 T cells (Figure 2A).

Phenotyping of the CD25^hi^Foxp3^hi^ and Foxp3^int^ populations on day 6 of co-culture (2∶1 iRBC∶PBMC ratio) showed a largely similar phenotype compared with conventional natural CD25^+^Foxp3^+^ Tregs (nTregs) in freshly isolated PBMCs (on day 0). All three populations expressed reduced levels of CD127 (IL-7R_alpha_) and CCR7, and enhanced levels of CTLA-4, CD122 (IL-2R_beta_), CD45RO, HLA DR and CD39 compared to CD4^+^CD25^−^ T cells in the same culture (Figure 2B). Interestingly, molecules associated with inhibitory function of nTregs in the literature such as CD39 and CTLA-4 were expressed at higher levels on these iRBC-induced Foxp3^+^ cells compared to nTregs. Additionally, induced day 6 Foxp3^hi^ and Foxp3^int^ CD4^+^CD25^hi^ T cells, but not day 0 nTregs, expressed high levels of ICAM-1, indicating recent activation. None of these markers did, however, help to distinguish between the CD25^hi^Foxp3^hi^ and CD25^hi^Foxp3^int^ cells. Two additional recently identified Treg-associated markers, ICOS [33]–[35] and TNFRII [36], were found in high level on induced Foxp3^hi^ and Foxp3^int^ cells. Expression of both molecules was slightly elevated on Foxp3^hi^ compared to Foxp3^int^ CD4^+^CD25^hi^ T cells, but both markers were equally unsuitable to purify the Foxp3^hi^ subset to analyse its suppressive capacity, as they did not distinguish clearly separate populations between the two subsets (Figure 2B).

### Th effector cytokine production by iRBC-induced Foxp3^int^ CD4 T cells is negatively affected by high ratios of Foxp3^hi^∶Foxp3^int^ cells

**Figure 3:**
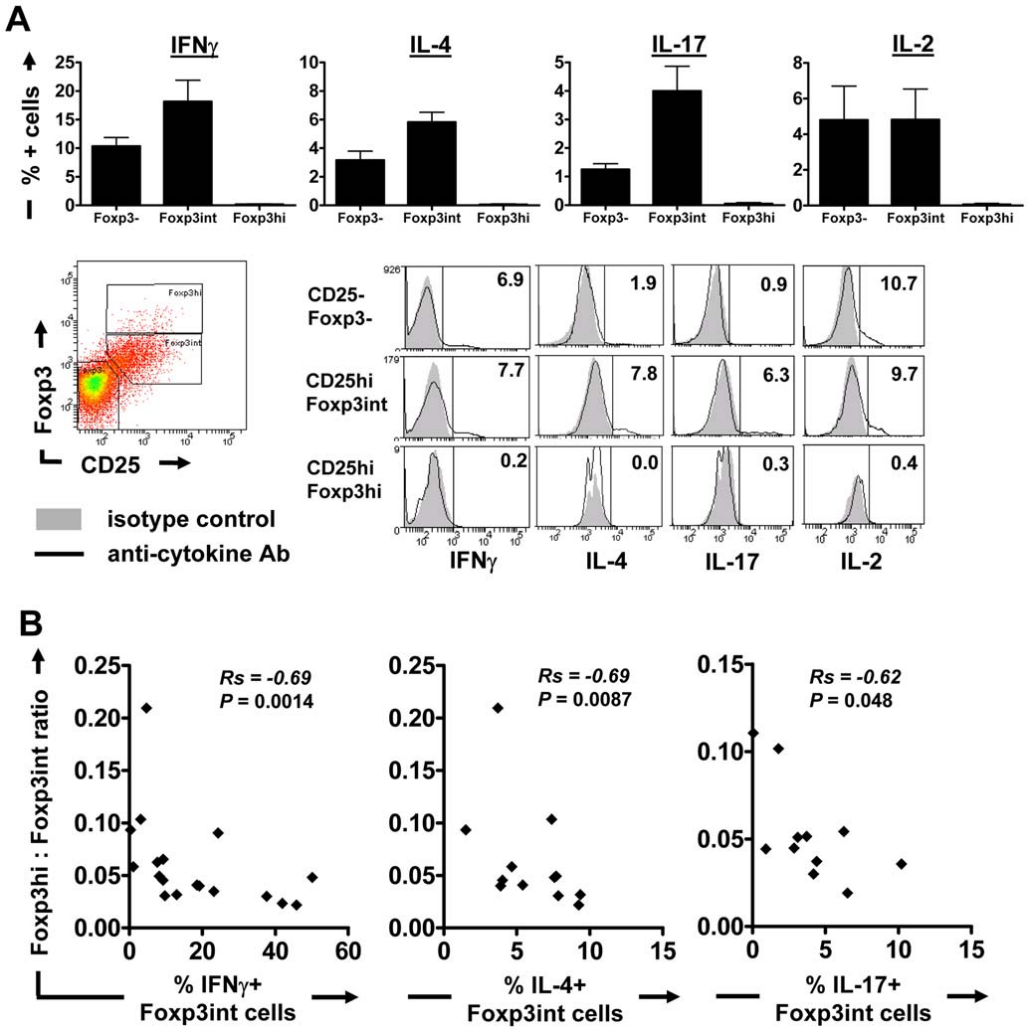
CD4^+^CD25^hi^Foxp3^hi^ T cells do not produce effector cytokines and high Foxp3^hi^∶Foxp3^int^ ratios inversely correlate with Th effector cytokine production by Foxp3^int^ cells. On day 6 of culture at an iRBC∶PBMC ratio of 1∶5, cells were re-stimulated for 5 hours with PMA and ionomycin, in the presence of brefeldin A for the last 4 hours. Surface marker and Foxp3 expression as well as cytokine production were analysed by flow cytometry. (A) Bar graphs show the percentage of IFNγ, IL-4, IL-17 and IL-2 producing cells within CD4^+^CD25^−^Foxp3^−^, Foxp3^int^ and Foxp3^hi^ CD4^+^CD25^hi^ T cells as Mean+/−SEM for all donors examined (n = 18 for IFNγ, n = 13 for IL-4, n = 11 for IL-17 and n = 5 for IL-2). Representative histogram plots show anti-cytokine staining (black line) versus isotype/background staining (grey filled) within each of the three gated populations. The percentage of cytokine positive cells within each gated population was determined using histogram plots as shown. (B) The relationship between Foxp3^hi^∶Foxp3^int^ ratios and the percentage of Th effector cytokine (IFNγ, IL-4, IL-17) producing cells within the CD25^hi^Foxp3^int^ population was examined using Spearman rank correlation.

To nevertheless determine whether Foxp3^hi^ and Foxp3^int^ CD4^+^CD25^hi^ T cells might differ functionally, cells were re-stimulated with PMA/ionomycin on day 6 of iRBC∶PBMC co-culture and examined for production of the pro-inflammatory T helper (Th) effector cytokines IFNγ, IL-4 and IL-17 as well as the T cell proliferation and survival factor IL-2 (Figure 3A). IL-2 was produced in similar amounts by both CD25^−^Foxp3^−^ and CD25^hi^Foxp3^int^ CD4 T cells (*P*>0.05), but not by CD25^hi^Foxp3^hi^ cells (*P*<0.05 compared to CD25^−^Foxp3^−^ and CD25^hi^Foxp3^int^ cells, one-way ANOVA with Neuman-Keuls' post-test). In contrast, the predominant Th effector cytokine producing cells in these cultures were the Foxp3^int^ population (*P*<0.001 for IL-17, *P*<0.01 for IL-4 and *P*<0.05 for IFNγ compared to CD25^−^Foxp3^−^ cells, and *P*<0.001 for IL-17, IL-4 and IFNγ compared to CD25^hi^Foxp3^hi^ cells, one-way ANOVA with Neuman-Keuls' post-test), indicating that these cells may constitute activated effector T cells. Finally, within the Foxp3^hi^ subset over 99% of cells did not produce any Th effector cytokines, which is consistent with the properties of Tregs.

We further utilized the combination of intracellular Foxp3 and Th effector cytokine staining as a means to gain insight into the potential suppressive activity of the Foxp3^hi^ population, whilst avoiding the necessity to purify the different subsets (which is not possible due to the lack of suitable surface markers to differentiate between the otherwise intracellularly stained Foxp3^hi^ and effector-like Foxp3^int^ cells). Specifically, we asked whether changes in the relative proportion of induced Foxp3^hi^ to Foxp3^int^ cells would affect the ability of the Foxp3^int^ population to produce Th effector cytokines. We concentrated on the Th1 and Th2 effector cytokines IFNγ and IL-4, which play important roles in anti-malarial immunity and immunopathology [37]–[40], as well as the Th17 cytokine IL-17, which promotes the recruitment of neutrophils [41] that contribute to the clearance of malaria-infected RBCs as well as malaria pathogenesis [42],[43]. If the Foxp3^hi^ population had indeed suppressive activity on the parasite-induced T effector population, then the Foxp3^int^ population in donors with higher ratios of Foxp3^hi^∶Foxp3^int^ cells would contain a smaller proportion of Th effector cytokine-producing cells compared to donors with a lower ratio of Foxp3^hi^∶Foxp3^int^ cells. Indeed, we found a strong and significant inverse correlation between the Foxp3^hi^∶Foxp3^int^ ratio and the proportion of cytokine-producing cells within the Foxp3^int^ population (Figure 3B; Spearman rank correlation; Rs = 0.69, *P* = 0.0014 for IFNγ; Rs = 0.69, *P* = 0.0087 for IL-4; Rs = 0.62, *P* = 0.048 for IL-17).

### iRBC-induced Foxp3^+^ CD4^+^ T cells have proliferated and are predominantly derived from the CD4^+^CD25^−^Foxp3^−^ T cell population

**Figure 4:**
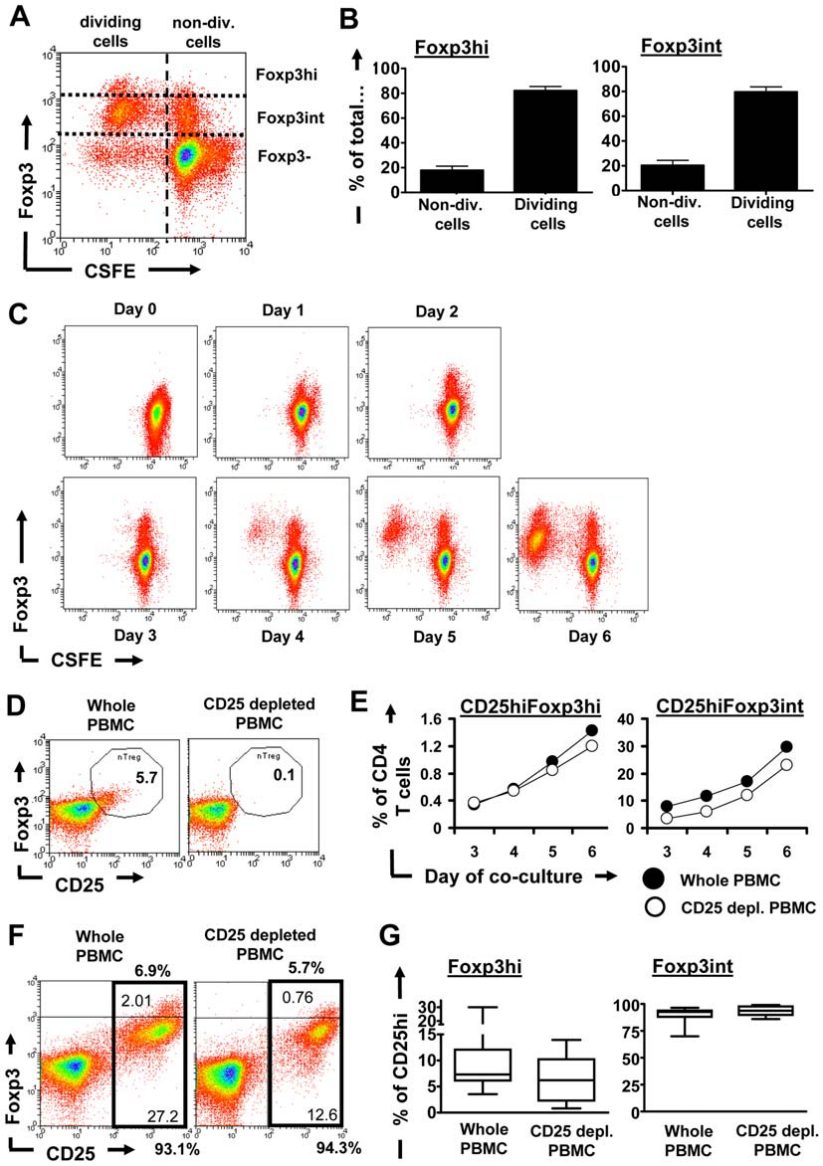
Expansion of iRBC-induced Foxp3^hi^ and Foxp3^int^ CD4^+^CD25^hi^ T cells from CD4^+^CD25^−^ and CD25^+^ T cells. PBMC were stained with CFSE prior to set up of iRBC∶PBMC co-cultures and analysed on day 6 (A). Data for dividing and non-dividing CD4^+^ T cells were analysed as percentage of total Foxp3^hi^ and Foxp3^int^ CD4^+^ T cells from 7 donors (Mean+/−SEM) (B). Representative FACS plots for 1 out of 3 donors show the development of Foxp3 expression and CFSE dilution over a time course of 6 days of iRBC co-culture (C) To determine the origin of Foxp3^hi^ and Foxp3^int^ CD4^+^CD25^hi^ T cells, PBMC were depleted from CD25^+^ cells prior to set up with iRBCs. A representative dot plot demonstrating depletion efficiency of the CD25^+^Foxp3^+^ nTreg population is shown (D). Cells were then analysed over a time course (day 3 to 6) for the kinetics of CD25 and Foxp3 induction by flow cytometry (E). A representative dot plot for 1 out of 15 donors showing the generation of CD25^hi^Foxp3^hi^ and CD25^hi^Foxp3^int^ on day 6 is shown. Percentages of both populations are expressed within total CD4 T cells (fine print) or within the CD25^hi^ population (bold print) (F). Due to a general decrease in the induction of total CD25^hi^CD4^+^ cells from CD25 depleted versus whole PBMCs, Foxp3^hi^ or Foxp3^int^ induction was analysed as percentage within the CD25^hi^ CD4 population. Data are represented as box-and-whisker-plots, with boxes extending from the 25^th^ to the 75^th^ percentile and horizontal lines representing the median, while whiskers extend to the lowest and highest data point (G). Experiments were set up with an iRBC∶PBMC ratio of 2∶1.

We next tested whether iRBC-induced Foxp3^hi^ and Foxp3^int^ cells were derived from proliferating CD4 T cells. PBMCs were labelled with CFSE and analysed on day 6 for CFSE dilution and Foxp3 expression within the CD4 T cell compartment (Figure 4A). As shown in Figure 4B, 79+/−4.1% of Foxp3^int^ and 82.2+/−3.4% of Foxp3^hi^ CD4 T cells (Mean+/−SEM) had proliferated within the 6 days of iRBC co-culture. When examining CFSE dilution and Foxp3 expression continuously over a time course of 6 days, we found that Foxp3 expression was initially up-regulated in the absence of proliferation, followed by proliferation resulting in a steady increase in both Foxp3^hi^ and Foxp3^int^ subsets (Figure 4C).

We next investigated whether the two Foxp3^+^ populations could be induced from CD25^−^ CD4 T cells, and whether there was a contribution from potentially expanding CD25^+^Foxp3^+^ CD4 nTregs present in the initial PBMC population. To answer this question, PBMCs were depleted of CD25^+^ cells prior to onset of the iRBC∶PBMC co-culture, with an average depletion efficiency of CD25^+^Foxp3^+^ nTregs by 93.8+/−2% (Mean+/−SEM) (Figure 4D). As shown in Figure 4E, CD25^hi^Foxp3^hi^ and CD25^hi^Foxp3^int^ were induced from both whole and CD25 depleted PBMC with similar kinetics. However, we found that in 11/15 donors, CD25 depletion prior to onset of iRBC∶PBMC co-culture led to a significant decrease (ranging from 6–72%, Figure 4F and data not shown) in the bulk iRBC-induced CD25^hi^ population compared to undepleted PBMC∶iRBC co-cultures. To determine the proportions of iRBC-induced Foxp3^hi^ or Foxp3^int^ cells from whole versus CD25 depleted PBMCs independent of the confounding influence of CD25 depletion on the total proportion of the induced CD4^+^CD25^hi^ T cells, we thus analyzed the percentage of Foxp3^hi^ and Foxp3^int^ cells within the CD25^hi^ compartment rather than within the complete CD4 T cell population. We found that overall there was a significant reduction in the iRBC-induced proportion of Foxp3^hi^ cells within the CD4^+^CD25^+^ T cell population in iRBC co-cultures with CD25 depleted PBMCs (Median 6.2%; interquartile range 2.3–10.2) compared to whole PBMCs (Median 7.3%; interquartile range 6.1–12.1; *P* = 0.015, Figure 4G). The induction of Foxp3^int^ cells, in contrast, was largely unaffected (Median 93.8% (interquartile range 89.8–97.7) from whole PBMCs compared to 92.7% (interquartile range 87.9–93.9) from CD25 depleted PBMCs). This indicates that the virtually all CD4^+^CD25^hi^Foxp3^int^ cells and the majority of CD4^+^CD25^hi^Foxp3^hi^ cells were derived from CD4^+^CD25^−^ non Tregs. However, CD25^+^Foxp3^+^ nTregs additionally contributed to the induction of CD4^+^CD25^hi^Foxp3^hi^ cells in iRBC∶PBMC co-cultures.

### Monocytes expressing increased levels of co-stimulatory molecules are sufficient for iRBC-mediated induction of Foxp3 expression

**Figure 5:**
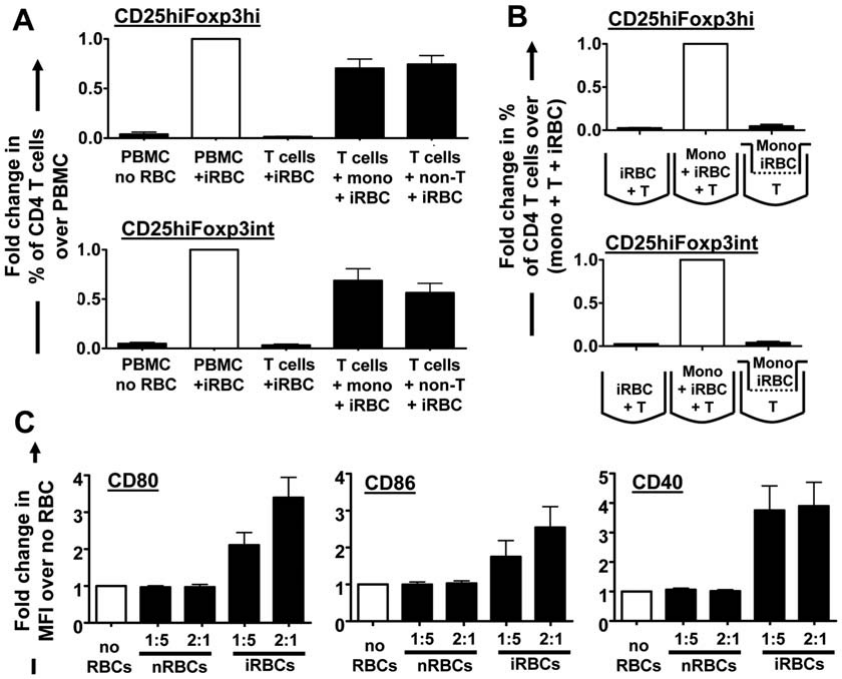
Monocytes are sufficient for Foxp3 induction. To determine the cellular requirements for Foxp3 induction, purified T cells were cultured with iRBCs alone or supplemented with donor-specific proportions of monocytes or all non-T cells and compared to iRBC co-cultured whole PBMCs (A). To determine contact-dependency, T cells were separated from monocytes through a transwell (B). Bar graphs show data of 7 (A) or 9 (B) donors (Mean+/−SEM) as relative proportions of CD25^hi^Foxp3^hi^ or CD25^hi^Foxp3^int^ cells. Fold changes were calculated by normalizing the percentage of each of the two cell types of CD4^+^ T cells for each donor on the percentage obtained for whole PBMC directly cultured with iRBCs (A) or direct monocyte+T cell+iRBC co-cultures (B). Experiments were set up with an iRBC∶PBMC ratio of 2∶1 and similar results obtained for a 1∶5 ratio. Levels of co-stimulatory molecules CD80, CD40 and CD86 on CD14^+^, CD3/19/56^−^ PBMC were analysed after 2 days of nRBC or iRBC∶PBMC culture as median fluorescent intensities (MFIs). Data were normalized for each donor on MFIs obtained for PBMCs cultured with no RBCs and are shown as Mean+/−SEM of 6–10 donors (C).

Foxp3 expression in CD4 T cells was not induced when purified T cells as opposed to whole PBMCs were co-cultured with iRBCs, demonstrating that induction of Foxp3^+^ CD4 T cells is not due to a direct interaction of T cells with iRBCs but dependent on other cells present in the co-culture system (Figure 5A). APCs have been suggested to play a fundamental role in the induction of Tregs [44], and monocytes are the most abundant APCs in human peripheral blood. To evaluate the possibility that monocytes interacting with iRBCs were responsible for the induction of Foxp3^+^ CD4 T cells, we co-cultured purified T cells with positively selected CD14^+^ monocytes at ratios consistent with the original proportions of T cells and monocytes in whole PBMCs determined for each donor. As shown in Figure 5A, CD14^+^ monocytes were sufficient to induce CD25^hi^Foxp3^hi^ and CD25^hi^Foxp3^int^ CD4 T cells. The levels of the two subsets induced were further similar to those observed when adding back all non-T cells including both monocytes and other PBMCs such as NK cells and B cells (obtained from negative T cell isolation). This demonstrates that the additional presence of other PBMCs did not have an additive effect on the levels of induced Foxp3^int^ or Foxp3^hi^ cells.

Monocytes further required direct contact with T cells to induce both CD25^hi^Foxp3^hi^ and CD25^hi^Foxp3^int^ CD4 T cells, as separation of T cells and monocytes through a transwell abrogated induction of Foxp3 expression (*P*<0.001, one-way ANOVA with Neuman-Keuls' post-test) (Figure 5B). When examining the activation status of CD14^+^ monocytes in iRBC∶PBMC co-cultures, we found that the levels of co-stimulatory molecules CD80, CD40 and CD86 were higher when iRBCs, but not nRBCs were added to the co-culture (*P*<0.01, one-way ANOVA with Dunett's post-test; Figure 5D), suggesting strong T cell co-stimulatory capacity. iRBC-mediated induction of CD80 and CD86, but not CD40 on monocytes was further dose-dependent, with lower expression levels at a 1∶5 compared to a 2∶1 iRBC∶PBMC ratio.

### iRBC-mediated induction of Foxp3 expression in converting CD4^+^ T cells requires MHC class II-induced soluble factors but not direct contact with monocytes

**Figure 6:**
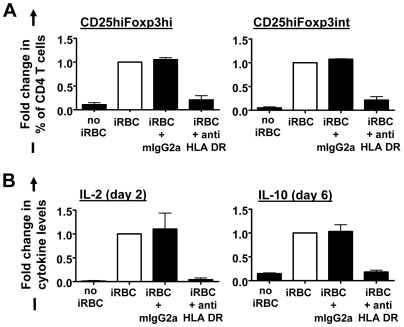
Foxp3 induction is MHC class II-dependent. MHC class-II dependency was examined using 5 µg/ml neutralizing anti-HLA DR or isotype control mAb. Cells were analysed for CD25 and Foxp3 expression on CD4 T cells on day 6 (A). Culture supernatants were sampled on day 2 (IL-2) or day 6 (IL-10) for cytokine analysis by ELISA (B). Fold changes in the proportion of Foxp3^hi^ and Foxp3^int^ cells (black bars) were calculated by normalizing for each donor the percentage of each of the two cell types of CD4^+^ T cells or cytokine levels on the respective values obtained for PBMCs cultured with iRBCs in the absence of any antibody (white bars). Experiments were set up with a 2∶1 iRBC∶PBMC ratio.

A possible molecular basis for the dependency of Foxp3 induction on contact between T cells and monocytes could be the interaction of monocyte-expressed MHC class II/peptide complexes with the TCR of CD4^+^ T cells. Indeed, blocking Abs directed against HLA DR (clone L243) inhibited the induction of Foxp3 expression (Figure 6A; *P*<0.001, one-way ANOVA with Neuman-Keuls' post-test). Blocking MHC class II-TCR interaction additionally severely reduced day 2 IL-2 levels and day 6 IL-10 levels in iRBC∶PBMC co-culture supernatants (*P*<0.01) (Figure 6B).

**Figure 7:**
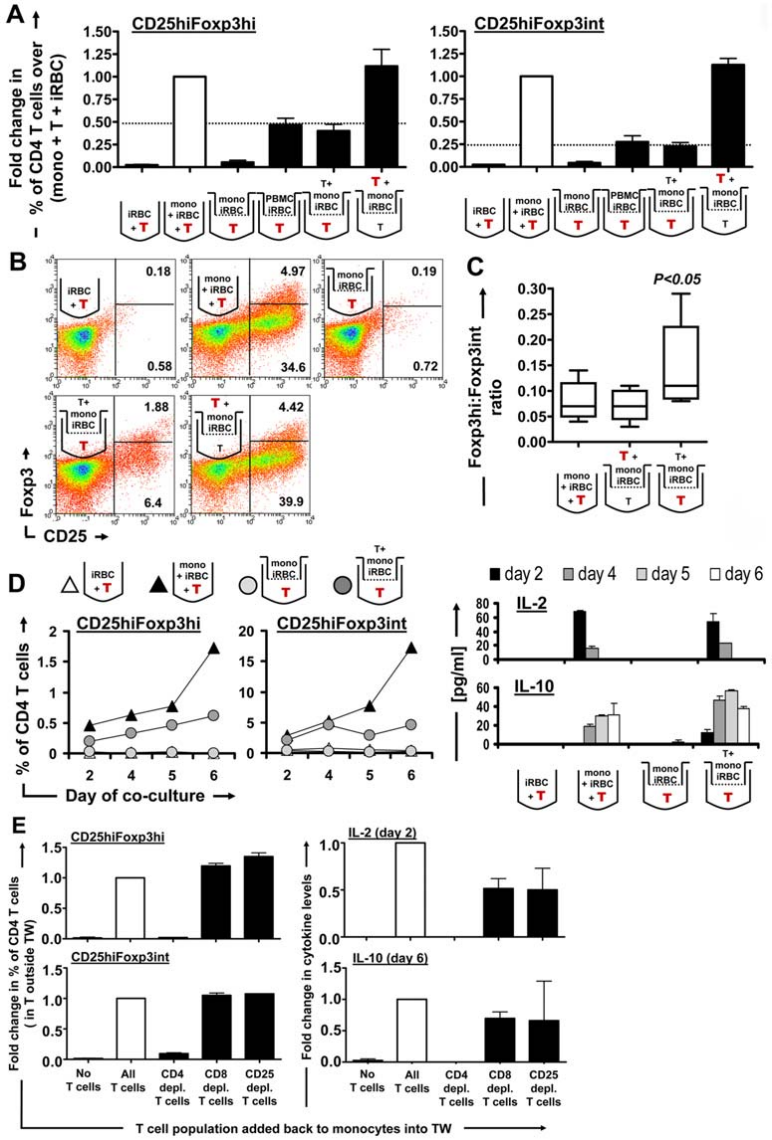
Foxp3 induction requires soluble factors from monocyte-CD4 T cell interaction, but not necessarily direct contact of monocytes with converting T cells. To determine contact-dependency for converting T cells, T cells were separated from iRBC-co-cultured whole PBMC or T cells+monocytes through a 20 nm pore size transwell and analysed by flow cytometry on day 6. Bar graphs show data of 5 donors (Mean+/−SEM), analysed as fold change in the induction of CD25^hi^Foxp3^hi^ or CD25^hi^Foxp3^int^ CD4 T cells (A). Representative FACS plots for each condition are shown (B). Foxp3^hi^∶Foxp3^int^ ratios were calculated for T cells in direct co-cultures as well as inside and outside the transwell (C). Foxp3^hi^ and Foxp3^int^ levels, as well as IL-2 and IL-10 levels in culture supernatants were monitored on day 2, 4, 5 and 6 and are shown for 1 out of 2 donors (D). To determine which T cell subpopulation has to interact with monocytes to induce Foxp3 expression in transwell-separated T cells, T cells that were added back to monocytes were depleted of CD4^+^, CD8^+^, or CD25^+^ cells prior to culture. Proportions of CD25^hi^Foxp3^hi^ and CD25^hi^Foxp3^int^ cells of CD4 T cells induced in the transwell-separated T cell compartment (on day 6) as well as IL-2 and IL-10 levels in the culture supernatant (on day 2 and 6, respectively) were measured and are shown as fold changes compared to whole T cells (E). Fold changes (black bars) were calculated by normalizing for each donor the percentage of each of the two cell types of CD4^+^ T cells or cytokine levels on the respective values obtained for monocytes+T cells directly cultured with iRBCs (A) or for cultures with whole T cells added back to monocytes within the transwell (E) (white bars). The T cell compartment analysed and depicted in each individual graph is highlighted in bold red. Experiments were set up with a 2∶1 iRBC∶PBMC ratio.

This raised the question whether the disruption of Foxp3 induction was due to the lack of MHC class II-induced soluble factors such as IL-2 and IL-10 produced by intermediate/bystander T cells, or to the lack of direct MHC class II interaction with the converting T cells themselves. To test these two possibilities, purified T cells were again separated from iRBC co-cultured monocytes through a transwell. Surprisingly, induction of CD25^hi^Foxp3^hi^ and to a lesser degree also CD25^hi^Foxp3^int^ CD4^+^ T cells outside the transwell could be partially restored when iRBC co-cultured monocytes inside the transwell insert were supplemented with T cells or replaced with iRBC co-cultured PBMCs (containing both T cells and monocytes) (*P*<0.001, one-way ANOVA with Neuman-Keuls' post-test; Figure 7A and B). There was no significant difference between the proportion of induced CD25^hi^Foxp3^hi^ and CD25^hi^Foxp3^int^ cells whether iRBC∶monocyte∶T cell co-cultures were set up inside or in the absence of a transwell (Figure 7A and B). Interestingly, Foxp3^hi^∶Foxp3^int^ ratios were significantly higher (*P*<0.05, one-way ANOVA with Neuman-Keuls' post-test) in CD4^+^CD25^hi^ T cells induced across the transwell in the absence of direct monocyte contact compared to direct iRBC∶monocyte∶T cells co-cultures either inside or in the absence of a transwell (Figure 7C). When examining the kinetics of CD25^hi^Foxp3^hi^ and CD25^hi^Foxp3^int^ cell induction in direct and transwell separated cultures, we found that whilst levels of CD25^hi^Foxp3^hi^ cell increased up to day 6 in both culture conditions, the proportion of CD25^hi^Foxp3^int^ CD4 T cells stagnated from day 4 onwards (Figure 7D). Both IL-2 and IL-10 were only detectable in supernatants from cultures where monocytes interacted with T cells (Figure 7D), consistent with the requirement of MHC/TCR contact for their induction (Figure 6B). This suggests that the direct interaction of iRBC-treated monocytes with bystander T cells via MHC class II induces soluble factors including IL-2 and IL-10, which in turn induce Foxp3 expression in CD4^+^ T cells that did not have direct contact with monocytes themselves.

We further examined which particular population of bystander T cells interacted with monocytes to promote the induction of Foxp3 expression across the transwell. We addressed this question by depleting purified T cells of anti-CD8, CD4 or CD25 single stained cells via FACS sorting to leave the T cells, which interact with the monocytes, untouched to avoid any possible interference of surface antibodies on T cells with their function. Depleted T cells were then added back to monocytes within the transwell at the original ratios as determined in whole PBMCs for each donor. As shown in Figure 7E, only CD8 depleted (i.e. CD4^+^ T cells), but not CD4 depleted (i.e. CD8^+^ T cells) interacting with monocytes within the transwell were capable of inducing Foxp3 expression across the transwell. In line with this, only the interaction of CD8 depleted (CD4^+^), but not CD4 depleted (CD8^+^) T cells with monocytes resulted in the secretion of IL-2 and IL-10 into the cell culture supernatant (Figure 7E), consistent with MHC class II dependency of these two cytokines as shown in Figure 6B. Depletion of CD25^+^ T cells did not decrease the induction of Foxp3 expression across the transwell, suggesting that nTreg interaction with monocytes is not required for Foxp3 induction on separated CD4 T cells.

**Figure 8:**
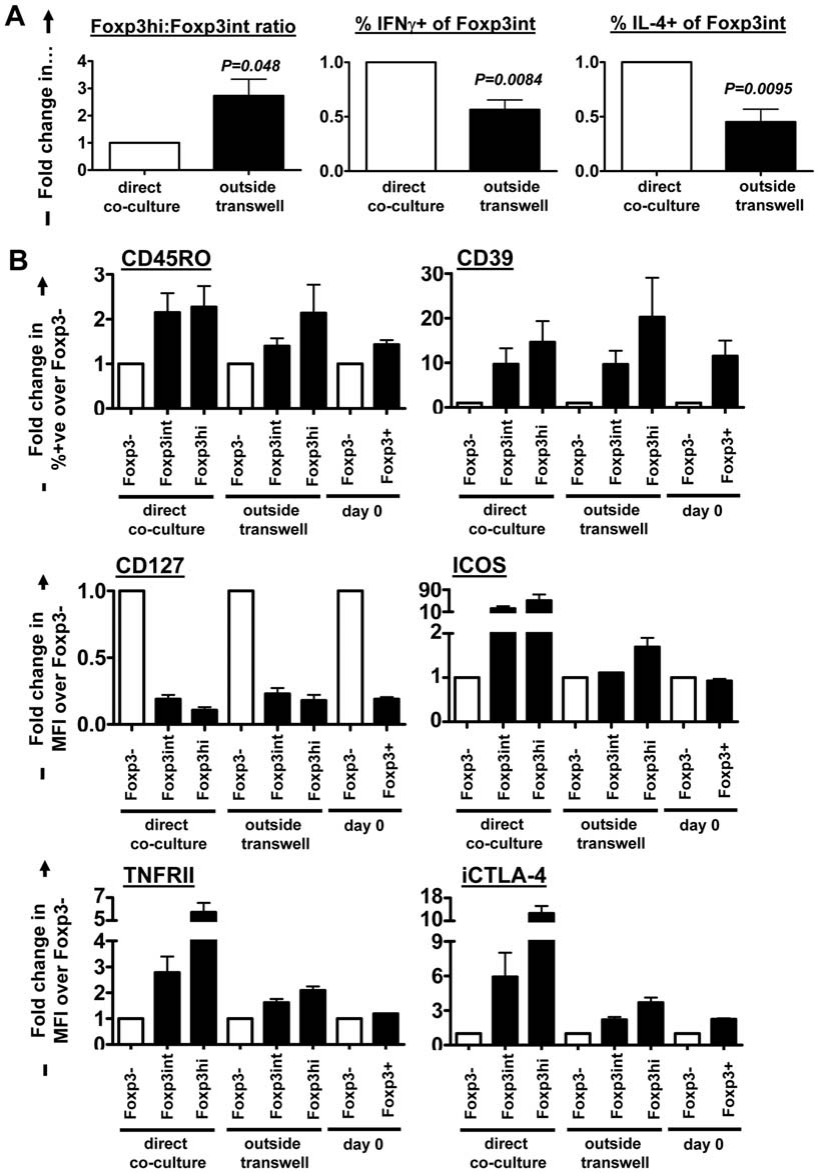
Phenotype and functional analysis of Foxp3^hi^ and Foxp3^int^ T cells in direct co-cultures or separated through a transwell. T cells from direct or transwell-separated iRBC co-cultures were harvested on day 6 of culture and either re-stimulated with PMA/ionomycin for intracellular cytokine staining, or phenotyped directly. Foxp3^hi^∶Foxp3^int^ ratios and the percentage of IFNγ or IL-4 producing cells within the CD25^hi^Foxp3^int^ population were determined for 5 donors. Values for transwell-separated T cells (black bars) are expressed as fold change following normalization onto values in direct co-cultures (white bars) for each donor (Mean+/−SEM) and compared by paired Student's *t*-test (A). Freshly isolated CD25^−^Foxp3^−^ and CD25^+^Foxp3^+^ CD4^+^ T cells (day 0) and CD4^+^CD25^−^Foxp3^−^, Foxp3^int^ and Foxp3^hi^ CD4^+^CD25^hi^ T cells from direct or transwell separated cultures (day 6) from 3 donors were phenotyped for a wide range of markers, and expression levels for each surface marker within each population were normalized on isotype control staining, when analysed as median fluorescence intensity (MFI). Expression levels of surface markers on CD4^+^CD25^−^Foxp3^−^ cells were comparable between conditions for each donor (data not shown). Thus, to compare the phenotype of day 0 CD25^+^Foxp3^+^ nTregs, and day 6 induced CD25^hi^Foxp3^int^ and CD25^hi^Foxp3^hi^ cells (black bars), expression levels are expressed as fold change (Mean+/−SEM) compared to CD4^+^CD25^−^Foxp3^−^ cells (white bars, relative level = 1) for each donor and condition (B). Experiments were set up with a 2∶1 iRBC∶PBMC ratio.

Finally, we asked whether CD25^hi^Foxp3^hi^ and CD25^hi^Foxp3^int^ cells generated in the transwell system independent of direct TCR stimulation displayed equivalent phenotypic and functional characteristics to the Foxp3^hi^ and Foxp3^int^ cells generated in direct iRBC∶PBMC cultures. Similar to direct co-cultures, only CD25^hi^Foxp3^int^ but not CD25^hi^Foxp3^hi^ cells contained a subset of Th1 and Th2 cytokine producing cells (data not shown). Moreover, transwell separated T cells showed both increased Foxp3^hi^∶Foxp3^int^ ratios and a decreased proportion of IFNγ and IL-4 producing cells within the Foxp3^int^ subset compared to CD4 T cells in direct co-cultures (Figure 8A), which is consistent with the results from the internal suppression assay shown in Figure 3B. We further analyzed the phenotype of Foxp3^hi^ and Foxp3^int^ cells induced in the direct co-culture or the transwell system and compared their phenotype to CD25^+^Foxp3^+^ nTregs from freshly isolated PBMCs prior to iRBC co-culture (Figure 8B). Induced CD25^hi^Foxp3^hi^ and CD25^hi^Foxp3^int^ cells in both culture systems had a very similar phenotype in regards to CD127, CD45RO and CD39 expression. TNFRII, ICOS and CTLA-4 expression was higher in Foxp3^hi^ compared to Foxp3^int^ cells in both direct and transwell-separated cultures, however, overall expression levels of these markers were 5–30 fold lower in Foxp3 expressing T cells induced in transwell-separated compared to direct cultures and more similar to day 0 nTregs.

### iRBC induce a pro-inflammatory cytokine milieu in combination with increased IL-10 and decreased TGFβ1 levels

**Figure 9:**
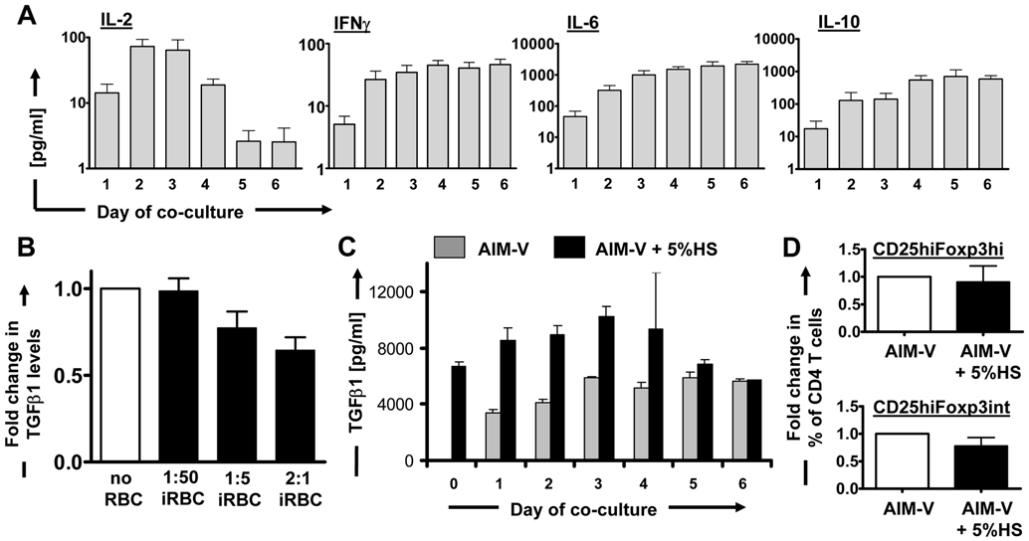
Cytokine levels in iRBC∶PBMC co-culture supernatants. Co-culture supernatants were harvested and assayed at different time points for IL-2, IFNγ, IL-6 and IL-10. Histograms show absolute values in pg/ml (Mean+/−SEM) for 14–18 donors (A). Total TGFβ1 levels in supernatants of untreated or iRBC-treated PBMC were determined on day 6 of co-culture and are shown as fold change for 12 donors, normalized on values from untreated PBMC (relative level = 1) for each donor (B). The role of serum-derived TGFβ1 for final TGFβ1 levels in co-culture supernatants was established in time course experiments for untreated (C) or iRBC-treated PBMC (data not shown) cultured in serum-supplemented or serum-free AIM-V. A representative histogram for supernatant TGFβ1 levels in pg/ml (Mean+/−StDev) for 1 out of 3 donors is shown (C). The proportions of Foxp3^hi^ and Foxp3^int^ CD25^hi^ CD4^+^ T cells in these cultures on day 6 were compared and are shown as Mean+/−SEM from 4 donors, normalized for each donor on values in serum-free cultures (D).

To further examine the role and nature of cytokines present in the co-culture system contributing to the induction of Foxp3 expression, we first characterized the iRBC-induced cytokine profile. As shown in Figure 9A, the pro-inflammatory cytokines IL-6 and IFNγ and the anti-inflammatory cytokine IL-10 were induced as early as 24 h after the addition of iRBCs and remained at high levels throughout the duration of the co-culture. IL-2 levels in the supernatant peaked at day 2–3 of co-culture and then declined to barely detectable levels on day 5 and 6 (Figure 9A). None of these cytokines were detectable in the absence of iRBCs.

High levels of total TGFβ1 (latent plus bioactive) were measured in the supernatant of untreated PBMCs on day 6 of culture, ranging from 0.6–13.5 ng/ml (23 donors; Median = 3.48 ng/ml; data not shown). Interestingly, day 6 TGFβ1 levels were significantly decreased when PBMCs were co-cultured with iRBCs (12 donors, *P*<0.01 for 2∶1 ratio and *P*<0.05 for 1∶5 ratio, one-way ANOVA with Neuman-Keuls' post-test; Figure 9B), but unchanged when PBMCs were co-cultured with uninfected RBCs (15 donors, data not shown). This decrease in total TGFβ1 was already evident and significant from 24 hours of co-culture onwards (data not shown) and dependent on the amount of iRBCs added to the co-culture (Figure 9B; *P*<0.0001, one-way ANOVA with linear trend post-test). A decrease in total TGFβ1 would be consistent with either increased conversion into bioactive TGFβ and immediate consumption, or decreased production and secretion. To test the latter, TGFβ1 mRNA levels for 2 donors were monitored over a time course of 6 days, showing no decrease in TGFβ1 mRNA in iRBC co-cultured PBMCs compared to control cultures, but instead a 2–3 fold increase on day 5–6 of co-culture (data not shown), suggesting a possible counter-regulatory mechanism to enhanced TGFβ consumption and therefore decreasing TGFβ protein levels in the cell culture supernatant. We next tested whether the high baseline levels of TGFβ1 in the iRBC∶PBMC co-cultures were due to autologous serum used to supplement the co-culture medium, or produced endogenously by cells within the co-culture system. While serum-supplemented medium did contain high levels of TGFβ1 before addition of cells (day 0), TGFβ1 levels increased over the first days of co-culture in both serum-supplemented and serum-free cultures, and reached comparable levels on day 6 of co-culture (Figure 9C). Further, there was no significant difference in the proportion of CD25^hi^Foxp3^hi^ and CD25^hi^Foxp3^int^ CD4^+^ T cells on day 6 of co-culture in serum-supplemented compared to serum-free iRBC∶PBMC co-cultures (Figure 9D), and no induction of these cells was seen in the absence of iRBCs as expected (data not shown). Foxp3 induction did therefore not depend on serum-derived exogenous TGFβ1, and TGFβ1 was produced by cells in the co-culture system.

**Figure 10:**
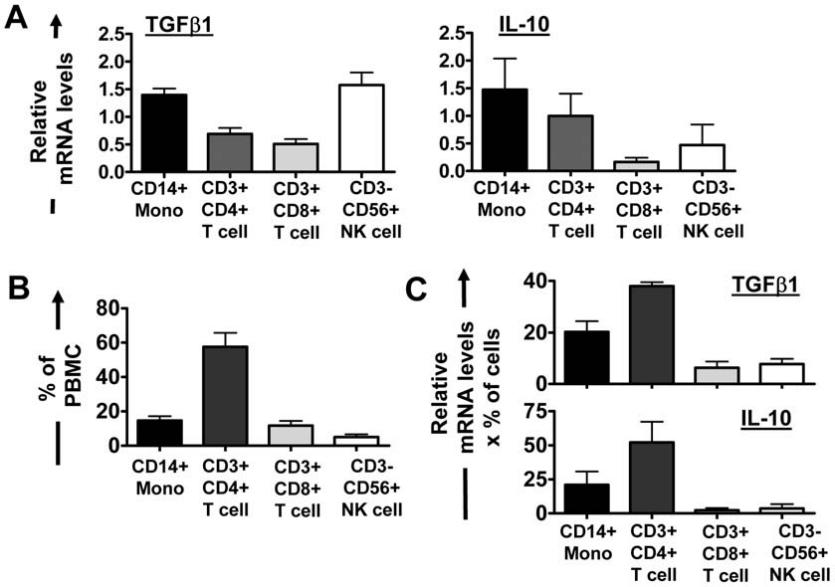
Monocytes and CD4 T cells are the main cellular source of TGFβ1 and IL-10 in iRBC∶PBMC co-cultures. On day 6 of iRBC∶PBMC co-culture (1∶5 ratio), individual cell types were separated by MACS and FACS to subsequently isolate RNA from purified populations. Relative mRNA levels (normalized on 18SrRNA) for TGFβ1 and IL-10 were determined and normalized for each donor on respective levels in total unseparated PBMC (A). The proportion of individual cell populations expressed as percent of total PBMC was determined by flow cytometry (B). Relative mRNA levels for IL-10 and TGFβ1 for each cell population were corrected for their proportion in whole PBMC by multiplying relative mRNA levels with the percentage of each population in whole PBMCs for each donor (C). Data (Mean+/−SEM) for 3 donors are shown.

To determine the cellular source of TGFβ1 and IL-10 in iRBC∶PBMC co-cultures, individual cell populations were FACS-sorted on day 6 and analysed for TGFβ1 and IL-10 mRNA levels expressed as normalized values compared to 18SrRNA, thus reflecting the average mRNA levels on a per cell basis. Across 3 donors tested, monocytes and CD3^−^CD56^+^ NK cells produced the highest amounts of TGFβ1 mRNA (Figure 10A; *P*<0.05 according to one-way ANOVA with Neuman-Keuls' post-test). Monocytes (as well as CD4 T cells) were also the major source of IL-10 mRNA (Figure 10A). Therefore, iRBC-exposed monocytes had an alternatively activated phenotype with increased levels of co-stimulatory molecules whilst at the same time producing immune suppressive cytokines. When correcting mRNA levels of each population for the proportion of this population in whole PBMCs (Figure 10B), however, CD4 T cells were the predominant source of cellular TGFβ1 and IL-10 mRNA in iRBC-treated PBMCs, closely followed by monocytes (Figure 10C). Moreover, in the absence of T cells, monocytes did not produce IL-10 upon iRBC exposure, and TGFβ1 levels were unchanged in the supernatant of purified monocytes co-cultured with either iRBCs or nRBCs, indicating a need for interaction with T cells to produce these cytokines (data not shown).

### IL-2 is required for Foxp3 induction in general, but only Foxp3^hi^ cells specifically require TGFβ and IL-10

**Figure 11:**
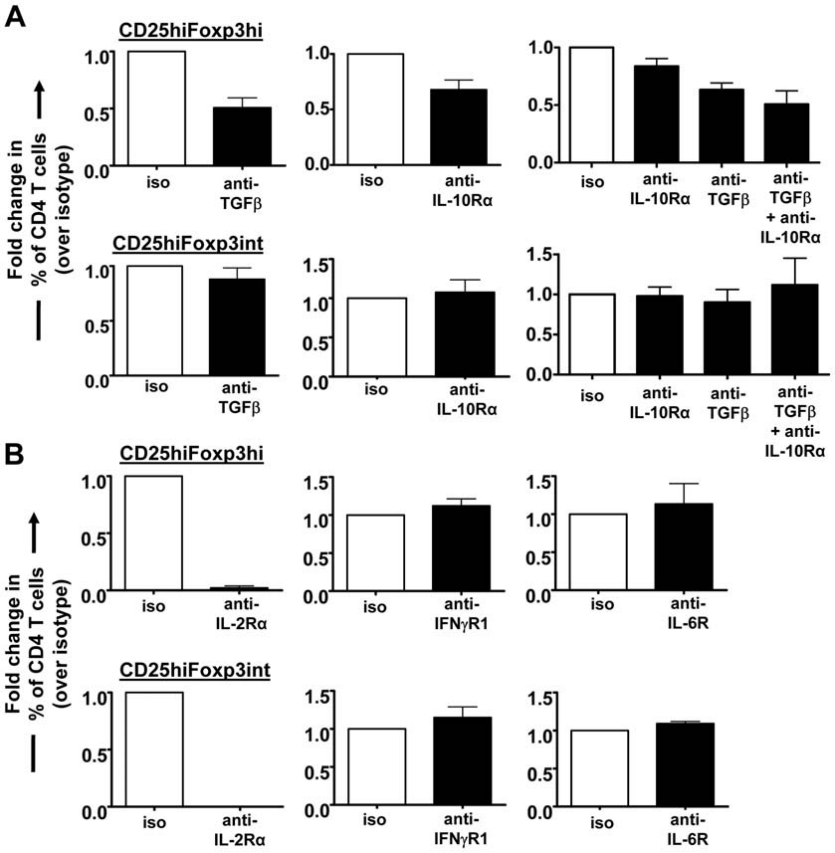
Differential role of TGFβ, IL-10 and IL-2 for the induction of Foxp3^hi^ and Foxp3^int^ T cells. The influence of neutralizing anti-TGFβ1,2,3 (100 µg/ml) and anti-IL-10R_alpha_ (50 µg/ml) alone or in combination (A), and receptor-blocking Abs for IL-2R_alpha_ (50 µg/ml), IFNγR_1_ (50 µg/ml) and IL-6R (5 µg/ml) (B) on the induction of Foxp3^hi^ and Foxp3^int^ CD4^+^CD25^hi^ T cells was determined by flow cytometry after 6 days of iRBC∶PBMC co-culture. Each graph shows data for 4–6 donors (Mean+/−SEM) as fold changes in the proportion of CD25^hi^Foxp3^hi^ or CD25^hi^Foxp3^int^ cells from total CD4 T cells. Fold changes were calculated by normalizing for each donor the percentage of each of the two cell types of CD4^+^ T cells in blocking/neutralizing Ab-treated cultures (black bars) on the respective percentage of CD4^+^ T cells obtained for isotype control-treated iRBC∶PBMC co-cultures (white bars).

To test whether TGFβ plays a role in the induction of Foxp3^+^ CD4 T cells by malaria-infected RBCs, we added TGFβ-neutralizing antibody to the iRBC∶PBMC co-culture. Neutralizing TGFβ reduced the proportion of induced CD25^hi^Foxp3^hi^, but not CD25^hi^Foxp3^int^ CD4 T cells down to 50.8+/−8% (Mean+/−SEM; *P* = 0.011) (Figure 11A). When assessing the effect of other blocking Abs, we found that blocking IL-10R_alpha_ also reduced the induction of Foxp3^hi^ cells (*P* = 0.035), but did not affect the Foxp3^int^ population. Combined treatment with anti-IL-10R_alpha_ and TGFβ-neutralizing antibodies in an additional set of 4 donors further decreased the induction of Foxp3^hi^ cells compared to anti-IL-10R_alpha_ treatment alone (*P*<0.05) but not compared to anti-TGFβ_1,2,3_ treatment alone, indicating synergistic action of the two cytokines but a major role for TGFβ compared to IL-10. Blocking IL-2R_alpha_, by contrast, severely inhibited the induction of both populations (inhibition >98%; *P*<0.001) consistent with their induction concomitantly with proliferation, while blocking the pro-inflammatory cytokine receptors IFNγR_1_ and IL-6R had no effect (Figure 11B). Foxp3^hi^ and Foxp3^int^ CD4^+^CD25^hi^ T cells were therefore induced by different mechanisms, requiring distinct cytokine environments.

## Discussion

CD4^+^CD25^+^Foxp3^+^ Tregs were initially thought to mainly play a role in immune homeostasis to avoid autoimmunity, but are increasingly also attributed roles in controlling immune responses to non-self antigens [8], thereby preventing immuno pathology, or alternatively contributing to immune evasion by tumors or invading pathogens [45]. An increase in peripheral CD4^+^CD25^+^Foxp3^+^ T cells has been observed in a number of infectious diseases including tuberculosis [46],[47], hepatitis C [48] and helminth infections [49],[50], as well as human [10]–[13] and murine [14]–[18] malaria infection. Current *in vitro* models examining the factors contributing to peripheral induction and expansion of human or murine CD4^+^CD25^+^Foxp3^+^ T cells combine strong TCR-stimulation using anti-CD3 mAb, allogeneic or peptide pulsed APCs together with exogenously added cytokines IL-2 and TGFβ [19], [20], [51]–[53].

In this study, we set out to determine whether the above signals also govern the induction of Foxp3 expression in human CD4 T cells by malaria-infected RBCs in the absence of any other exogenous stimuli. We set up an *in vitro* co-culture system of freshly isolated human PBMCs and trophozoite-stage iRBCs at a range of ratios reflecting parasitemias during human malaria infection. This co-culture resulted in the iRBC concentration-dependent induction of CD4^+^CD25^hi^ IL-10 and TGFβ1 mRNA-producing T cells. These induced CD4^+^CD25^hi^ T cells could be further subdivided by expression of Foxp3 at either high or intermediate levels. iRBC-induced CD4^+^CD25^hi^Foxp3^hi^ and Foxp3^int^ T cells had a phenotype reminiscent of typical CD4^+^CD25^+^ natural Tregs, including the expression of immuno-suppressive mediator molecules such as CTLA-4 or CD39 [23]. In contrast to natural Tregs, they did, however, also express markers indicating recent activation. CD4^+^CD25^hi^Foxp3^hi^ T cells additionally expressed higher levels of the Treg markers ICOS and TNFRII compared to Foxp3^int^ T cells. In line with this, Walther and co-workers recently also found a correlation between Foxp3 and TNFRII levels on Tregs from malaria infected children [13], and Minigo and colleagues reported higher levels of TNFRII expression on Tregs from individuals with severe as opposed to uncomplicated malaria [12]. Out of the parasite-induced CD25^hi^ CD4 T cells, the Foxp3^hi^ population was practically devoid of pro-inflammatory cytokine producing cells consistent with a T regulatory phenotype. In contrast, the Foxp3^int^ population contained significant subsets of cells producing the major Th effector cytokines IFNγ (Th1), IL-4 (Th2), IL-17 (Th17) as well as the T cell growth factor IL-2 consistent with an effector phenotype. The predominant cytokine produced by the Foxp3^int^ subset was the Th1 cytokine IFNγ, consistent with a recent study by Walther and co-workers [13]. Moreover, we found a significant inverse correlation between Foxp3^hi^∶Foxp3^int^ ratios and the percentage of IFNγ, IL-4 and IL-17 producing cells within the Foxp3^int^ population, indicating suppressive activity of parasite-induced Foxp3^hi^ cells on Th effector cytokine production by parasite-induced effector Foxp3^int^ cells. Interestingly and in line with our findings, Treg frequencies determined by Foxp3 mRNA levels were recently shown to negatively correlate with the magnitude of malaria-specific memory IFNγ responses in human malaria patients [13]. Moreover, an ethnic group in West Africa, the Fulani, shows a functional deficit in Treg related gene expression [54], as well as elevated levels of peripheral blood malaria-specific Th1 (IFNγ) and Th2 (IL-4) producing cells [55].

The majority of both CD4^+^CD25^hi^Foxp3^hi^ and Foxp3^int^ T cells had proliferated and were also induced in iRBC∶PBMC cultures previously depleted of natural CD4^+^CD25^+^ Tregs, strongly suggesting their main origin in the CD4^+^CD25^−^ naïve T cell population, with an additional and independent contribution from natural Tregs to the parasite-induced generation of CD4^+^CD25^hi^Foxp3^hi^ T cells. Future studies may further explore the potentially different functional properties or alternative pathways of generation of the subset of Foxp3^+^ T cells that failed to proliferate by day 6 of co-culture. Monocytes expressing increased levels of co-stimulatory molecules as well as IL-10 and TGFβ mRNA were sufficient for iRBC-induced Foxp3 expression in purified T cells.

**Figure 12:**
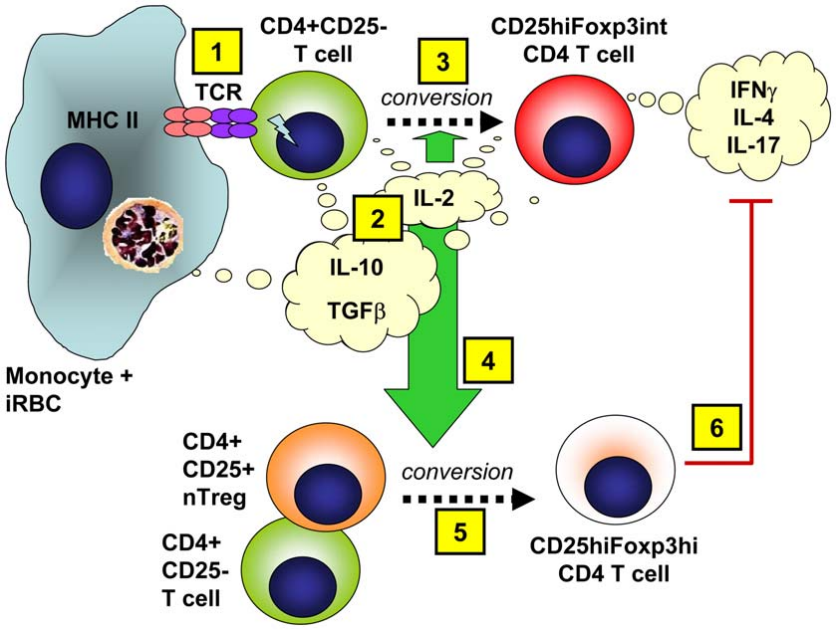
Proposed model of Foxp3 induction by malaria-infected RBCs. Upon exposure to malaria-infected RBCs, monocytes interact with CD4^+^ T cells via MHC class II (1), which induces IL-2 and IL-10 secretion from these cells (2). IL-2 contributes to the conversion of CD4^+^CD25^−^ into CD25^hi^Foxp3^int^ cells (3). Additionally, CD4^+^ T cell-derived IL-2 in conjunction with monocyte and T cell-derived IL-10 and TGFβ acts on bystander CD4^+^CD25^−^ T cells as well as CD4^+^CD25^+^ nTregs, that have not interacted with monocytes and therefore received no signal through their TCR themselves (4). This drives/enhances Foxp3 expression in these cells, resulting in the generation of CD25^hi^Foxp3^hi^ CD4 T cells (5). These CD25^hi^Foxp3^hi^ CD4 T cells in turn regulate the production of the Th effector cytokines IFNγ, IL-4 and IL-17 by parasite-induced CD25^hi^Foxp3^int^ CD4 T cells (6).

Importantly we demonstrate here for the first time, to our best knowledge, that direct TCR stimulation is not strictly required for Foxp3 induction in CD4 T cells by an infectious agent. The induction of Foxp3 expression occurred in transwell-separated responder/bystander T cells solely responding to soluble factors without their direct interaction with monocytes. The 20 nm pore size of the transwell inserts also eliminates the possibility that monocyte derived MHC class II-bearing exosomes, which are 50–100 nm in diameter, could have transferred antigen between the two chambers [56],[57]. A final possibility would be TCR stimulation resulting from direct T cell-to-T cell presentation of iRBC-derived peptides, possibly acquired from APC-derived soluble MHC as reported for CD8^+^ T cells [58]. This possibility can, however, also be excluded as Foxp3 induction did neither occur when T cells were directly cultured with iRBCs, nor when T cells were separated from iRBC-exposed monocytes, as a potential source of such peptides, through a transwell. Instead, monocyte/MHC class II interaction with helper CD4 T cells was critical to initiate the secretion of the soluble mediators IL-2 and IL-10, which were necessary for the induction of Foxp3^hi^ expression in transwell-separated bystander CD4^+^ T cells (Figure 12). The generally lower levels of either Foxp3^int^ or Foxp3^hi^ T cells outside the transwell (compared to directly cultured cells) can at least partially be explained by the consumption of cytokines as well as other potential soluble mediators by CD4 T cells inside the transwell insert, converting into Foxp3^+^ cells themselves. While we cannot exclude that soluble factors derived from TCR-stimulated T cells inside the transwell might additionally act in an autocrine fashion in concert with TCR-stimulation in these cells, this was clearly not the case for their counterparts outside the transwell.

These findings contrast the common assumption that peripheral induction of Foxp3 expression in CD4^+^CD25^−^ T cells necessarily requires stimulation through the TCR [20]. While it has been shown that the *foxp3* gene has response elements that can positively regulate the trans activation of the *foxp3* promoter after triggering of the TCR [59]–[61], direct TCR stimulation through APCs, according to our data, is not an absolute requirement. The fact that the induction of Foxp3 expression in CD4^+^ T cells does not require direct contact of these cells with APCs such as monocytes via the TCR also implies that there is no mechanism to ensure that induction of Foxp3 expression is confined to T cells specific for the same antigen, as proposed elsewhere [20]. This in turn may explain why immune suppression during acute malaria infection, for instance, is not confined to T cell responses against malarial antigens, but also other pathogens such as Epstein Barr virus [5],[62], Herpes Zoster virus [3] or *Salmonella* species [6] as well as vaccine antigens administered during and after acute malaria infections [4],[7].

CD25^hi^Foxp3^hi^ and CD25^hi^Foxp3^int^ cells generated outside the transwell without direct monocyte contact did further display similar functional characteristics compared to those induced in direct co-cultures, and similar expression levels of CD127, CD39 and CD45RO compared to day 0 CD25^+^Foxp3^+^ nTreg and day 6 Foxp3^hi^ and Foxp3^int^ cells in direct co-cultures. However, while TNFRII, ICOS and CTLA-4 expression was higher in Foxp3^hi^ compared to Foxp3^int^ cells in both direct and transwell-separated cultures, overall expression levels of these markers were markedly lower in Foxp3 expressing T cells induced in transwell-separated cultures, indicating that high levels of expression of these molecules do require direct TCR or co-stimulation of these cells. In fact all three molecules are known to be up-regulated upon TCR stimulation, and CD80/CD86-mediated CD28 co-stimulation of T cells further increases CTLA-4 expression and is required for ICOS expression [63]–[66]. Thus, while Foxp3^hi^ with a Treg phenotype and functional capacity, and to a lesser extend also effector-like Foxp3^int^ cells, can be induced across the transwell by soluble factors in a TCR independent manner, it is likely from these data that in direct co-cultures these cells do in fact receive a TCR/co-stimulatory signal that enhances expression levels of some, but not all surface markers including TNFRII and ICOS as well as intracellular CTLA-4.

Foxp3^hi^∶Foxp3^int^ ratios were higher in transwell separated T cells, most likely due to the stagnation of Foxp3^int^ levels but a further increase in Foxp3^hi^ levels from day 4 onwards. This suggests that the induction/expansion of Foxp3^int^ cells may be more reliant on cell-to-cell contact, while Foxp3^hi^ CD4^+^ T cells may be more easily induced and expanded by soluble factors in the co-culture system. Specifically, we found that both populations critically relied on monocyte/MHC class II-induced IL-2, which is consistent with the multiple roles of IL-2 in regulatory and inflammatory T cell responses [67]. However, only CD25^hi^Foxp3^hi^ CD4 T cells, which were detectable 24 later than CD25^hi^Foxp3^int^ cells, were also dependent on TGFβ1 and IL-10. Interestingly, IL-10 and TGFβ secreted by natural or induced Treg have been reported to convert naive CD4 T cells into Foxp3 expressing Tregs [68]. The importance of these two cytokines for the induction of CD4^+^CD25^hi^Foxp3^hi^ T cells may explain previous findings showing an association between IL-10 and TGFβ production early during malaria infection and an increase in CD4^+^CD25^+^(Foxp3^+^) T cells [11],[12],[28]. Further, CD4^+^ T cells, and specifically those in the CD25^hi^ fraction, were the major cellular source of these two cytokines in iRBC-co-cultured PBMCs. In this context it was recently shown that effector T cells co-producing IFNγ and IL-10, which are more prevalent in children with mild than severe malaria, may help to regulate acute malarial inflammation [13]. It is therefore a possibility that the more contact-dependent CD4^+^CD25^hi^Foxp3^int^ T cells constitute the above mentioned TCR-stimulated, cytokine secreting helper T cells (which via IL-2, IL-10 and potentially TGFβ secretion subsequently act on responder/bystander T cells), thereby further promoting TCR-independent induction of CD4^+^CD25^hi^Foxp3^hi^ T cells (summarized in Figure 12). Future studies using this *in vitro* iRBC∶PBMC co-culture system can further investigate the potential roles of mediators other than MHC class II stimulation and TCR stimulation or the above cytokines in the induction of Foxp3 expression.

Besides CD4^+^CD25^hi^ T cells, monocytes in iRBC∶PBMC cultures were a major source of the immuno modulatory cytokines IL-10 and TGFβ1 in our study. This is in accordance with an earlier study on Treg induction in experimental malaria infection of healthy UK volunteers, which identified monocytes as the predominant source of early serum TGFβ [11]. IL-10 production has equally been attributed to iRBC-exposed APCs [69]–[72]. Interestingly, however, we found no detectable IL-10 in the supernatant of purified monocytes co-cultured with iRBCs in the absence of, or transwell-separated from T cells, and there was also no difference in supernatant TGFβ levels between purified monocytes co-cultured with iRBCs or nRBCs (data not shown). This indicates that contact with T cells may be required for monocytes to produce these cytokines in response to iRBC exposure, and future studies are needed to further explore the interplay and potential reciprocal modulation between the two cell types.

iRBC-exposed monocytes also up-regulated expression of co-stimulatory molecules, which for CD80 and CD86 was dependent on the parasite dose. In pregnancy-associated malaria, iRBCs preferentially accumulate in the placenta leading to higher parasite exposure of placental compared to peripheral blood monocytes [73], and CD80 and CD86 expression was higher in placental compared to peripheral monocytes [74]. The contribution of diverse parasite factors and their corresponding receptors on monocytes to monocyte activation and subsequent induction of Foxp3 expression will be an important subject of future studies and may involve molecules such as TLR9 [75] and the scavenger receptor CD36 [76]–[78]. The induction of Foxp3 expression in CD4^+^ T cells by APCs expressing the co-stimulatory molecules CD80 and CD86 is consistent with previous reports [53],[79]. CTLA-4 ligation via CD80 shortly after T cell activation is necessary to enable TGFβ-induced Foxp3 expression [80], and co-stimulation via CD28 has been shown to mediate survival of murine Tregs induced by TCR/TGFβ1-stimulation [81]. Banerjee and co-workers further demonstrated that APCs are most effective in inducing functional Foxp3^+^CD4^+^ T cells when treated with pro-inflammatory cytokines TNF, IL-6 and IL-1β [79], all of which were detectable in the iRBC∶PBMC co-cultures used in this study. Importantly, co-stimulation by APCs has been found to be necessary to induce T cell-derived IL-2, which is crucial for the induction of Foxp3 expression [44],[53] and consistent with the findings presented here. IL-2 does not, however, directly induce Foxp3, but is essential for TGFβ1 induced Foxp3 expression in CD4^+^CD25^−^ T cells as well as their expansion [51]. Furthermore, IL-2 signaling via STAT5 through the IL-2Rβ chain (CD122), which is expressed by both Foxp3^hi^ and Foxp3^int^ CD4^+^CD25^hi^ T cells in this study, has been shown to be crucial for Foxp3 expression and Treg function [82],[83].

The fact that Foxp3 induction also occurred in iRBC∶PBMC cultures previously depleted of natural CD4^+^CD25^+^ Tregs is consistent with the origin of future Foxp3^+^ cells in the CD4^+^CD25^−^ naïve T cell population and supported by other studies describing this phenomenon [53], [84]–[86]. IL-2 dependent induction of Foxp3 in this population devoid of IL-2 receptor expression can be explained by the coordinated induction of CD25 by the parasite cytokine environment: IL-6, TNFα and TGFβ for instance are all known to induce CD25 expression [66], [84]–[89], and IL-2 itself has been shown to promote gene expression of its own receptor [90] even in human CD25 depleted PBMC, i.e. in the absence of IL-2 receptor expression, on both CD4^+^ and CD8^+^ T cells in the absence of antigenic stimulation [91]. Thus, cytokines including TGFβ and IL-2 as well as potential parasite-derived factors in iRBC∶PBMC co-cultures may induce CD25 expression in naïve CD4^+^CD25^−^ T cells, rendering these cells responsive to IL-2, resulting subsequently in enhanced CD25 expression, IL-2 promoted Foxp3 induction and proliferation.

Decreased TGFβ1 levels in the co-culture supernatants are consistent with increased consumption of TGFβ and were evident as early as 24 hours after addition of iRBCs to PBMCs. The importance of early TGFβ exposure for Foxp3 induction has been shown *in vitro* [52] and in experimentally malaria-infected human volunteers [11]. Furthermore, it has been observed that Treg-derived surface TGFβ or TGFβ bound to latency associated peptide on the surface of induced Tregs [20] or immature DCs [92] can in turn induce Foxp3 expression in responder cells. The Fulani, an ethnic group in West Africa with a decreased susceptibility to *P. falciparum* malaria, have a deficit not only in Foxp3 but also TGFβ, TGFβR mRNA expression in CD4^+^CD25^+^ Tregs and lower serum TGFβ levels [54]. This raises the question whether insufficient TGFβ availability might contribute to the deficit in Foxp3 in these cells and hence the more potent anti-malarial immune responses in this population.

In summary, we examined and defined the contribution of host immune mechanisms including APC-mediated TCR stimulation and cytokines to the induction of Foxp3 expression by the malaria parasite. Using an *in vitro* co-culture system of purified parasitized RBCs and PBMCs in the absence of any artificial exogenous stimuli, we have demonstrated the *in vitro* generation of two distinct populations of effector-like Foxp3^int^ and regulatory cell-like Foxp3^hi^ CD4^+^CD25^hi^ T cells. Our co-culture system may well reflect the processes occurring *in vivo* during malaria-infection, and explain the elevated levels of CD4^+^CD25^hi^Foxp3^+^ T cells observed in malaria-infected individuals and the association of IL-10, TGFβ and IL-2 with their induction. The induction of these cells even in the absence of direct contact of monocytes with converting T cells implies that peripheral induction of CD4^+^CD25^+^Foxp3^+^ cells is, in contrast to current belief, not necessarily linked to direct antigen-specific TCR stimulation. Future studies are now required to determine whether other pathogens are also able to induce such cells without the need for direct and concomitant TCR stimulation.

## Materials and Methods

### *P. falciparum* culture and trophozoite isolation

Mycoplasma-free blood-stage parasites of *Plasmodium falciparum* (strain 3D7) were maintained in O^+^ erythrocytes in RPMI-1640 medium (JRH, Lenexa, KS, USA) supplemented with 1 mM glutamine, 11 mM glucose, 25 mM HEPES, 0.2% (w/v) sodium bicarbonate, 200 µM hypoxanthine, 40 µg/ml gentamycin (all Sigma-Aldrich, St. Louis, MO, USA), and 0.5% (w/v) AlbuMAX II (GIBCO, Invitrogen, Carlsbad, CA, USA) at 37°C in an atmosphere of 5% CO_2_ and 1% O_2_ in N_2_. Knob-expressing parasites were enriched weekly using gelofusine solution (Braun Melsungen, Germany). Trophozoite stage parasites were isolated by density gradient centrifugation following layering onto a gradient of 40/60/80% isotonic Percoll (Amersham Biosciences, Uppsala, Sweden). The percentage of infected erythrocytes was typically 90–100%.

### PBMC isolation and iRBC∶PBMC co-culture

PBMCs were recovered by Ficoll–Hypaque (Amersham Biosciences) density gradient centrifugation from buffy coats (Australian Red Cross Blood Service, Melbourne, Australia). Autologous human serum (HS) was obtained by coagulating platelet-rich plasma from buffy coats with 0.3% (w/v) CaCl_2_, followed by heat-inactivation at 56°C for 30 min. PBMCs were cultured in AIM-V medium (GIBCO, Invitrogen) supplemented with 5% autologous HS alone (untreated controls), with non-infected control erythrocytes or trophozoite-stage iRBCs. iRBC∶PBMC ratios were calculated to reflect parasitemia ranges in natural infections (2∶1 ratio ∼0.1% parasitemia or 5000 iRBC/µl blood; 1∶5 ratio ∼0.01%; 1∶50 ratio ∼0.001%), assuming an average RBC count of 6×10^6^/µl blood and 3×10^3^ PBMCs/µl blood (white blood cell count minus granulocytes). In some experiments, tissue culture inserts (Anopore, NUNC, Naperville, IL, USA) were used to separate iRBC-treated (inside) and untreated cell populations (outside the transwell). A pore size of 20 nm was chosen to prohibit transfer of hemozoin crystals, which are on all sides larger than this cut-off [93].

### Magnetic cell isolation and add-back experiments

PBMCs were separated using the Pan-T cell negative isolation kit and CD14 microbeads (Miltenyi Biotech, Bergisch Gladbach, Germany) according to the manufacturer's recommendations in combination with LS (negative selection), MS (positive selection) and LD (depletion) columns. T cells and monocytes were typically >97% pure. CD14^+^ monocytes were added back to purified T cells based on original donor-specific ratios as determined by flow cytometry analysis for each donor. CD3^+^ T cells ranged from 45–57% and CD14^+^ monocytes from 7–31% of total PBMCs. For some experiments, PBMCs were depleted of CD25^+^ cells using anti-CD25 PE antibody (BD Pharmingen) and anti-PE microbeads (Miltenyi Biotech).

### Cell phenotyping by flow cytometry and intracellular cytokine staining

Anti-CCR7 PE was purchased from R&D systems (Minneapolis, MN, USA), and anti-CD39 PE was from Serotec (Oxford, UK). Anti-Foxp3 (clone PCH101) and anti-IL-17A PE were obtained from eBiosciences (San Diego, CA, USA) and anti-Foxp3 (clone 259D) was from Biolegend (San Diego, CA, USA). Anti-IL-2 APC was from Miltenyi Biotech. All other fluorochrome-conjugated monoclonal antibodies and isotype controls were purchased from BD Pharmingen (San Jose, CA, USA). For intracellular cytokine staining, cells were re-stimulated on day 6 of iRBC∶PBMC co-culture for 5 hours with phorbol 12-myristate 13-acetate (PMA) (50 ng/ml) and ionomycin (1 µg/ml) (both Sigma) in the presence of brefeldin A (eBioscience) for the last 4 hours. Cells were washed with PBS and incubated with antibodies diluted in PBS/10% HS/0.01% NaN_3_ (sodium azide) for 30 min on ice. Intracellular staining for Foxp3, CTLA-4 and cytokines was performed using the eBioscience fixation/permeabilization buffer kit. A minimum of 10^5^ events in the lymphocyte gate was acquired using a FACScalibur flow cytometer for 4-colour analysis and on a FACSAria for 5 to 6-colour analysis, and analyzed using WEASEL software (WEHI, Melbourne, Australia). Cells were gated first based on forward and side scatter to excluded dead cells and cell debris. Cells in the lymphocyte gate were then further gated based on forward scatter (to exclude monocytes) versus CD4 expression. Whenever possible, a CD3 stain was included as well to ensure that gated CD4^+^ cells were indeed T cells. Phenotyping of monocytes was carried out by gating on CD14^+^ cells that were negative for other lineage markers (CD3, CD19, CD56). All histograms and dot plots are depicted in log scale.

### CFSE proliferation

CFSE labeling was employed to distinguish between dividing and non-dividing cells [94]. Freshly isolated PBMCs were re-suspended at 10^6^cells/ml in PBS/0.1% FCS and incubated with 0.4 µM CFSE (Molecular Probes, Invitrogen) for 5 minutes at 37°C before staining was quenched with ice-cold RPMI/10% FCS. Cells were washed and set up as described above.

### Inhibition assays with anti-MHC class II and cytokine receptors-blocking antibodies

To examine the effect of cytokines, PBMCs were pre-incubated for 1 h at 37°C with blocking antibodies directed against human IFNγR_1_ (mIgG_1_, clone 92101), IL-2R_alpha_ (mIgG_1_, clone 22722), IL-6R (polyclonal goat IgG), IL-10R_alpha_ (mIgG_1_, clone 37607) or TGFβ1,2,3 (mIgG_1_, clone 1D11) (all R&D Systems). MHC class II dependency was determined using anti-HLA DR mAb (mIgG_2a_, clone L243; BD Pharmingen). Normal goat IgG, mIgG_1_ or mIgG_2a_ were used as isotype controls. All antibodies were sodium azide free. iRBC-PBMC co-cultures for each donor were set up in triplicate.

### Assays for cytokines

Supernatants were collected at indicated time points and stored at −20°C until assayed in duplicate for cytokines using Cytokine ELISA OptEIA kits (BD Pharmingen) according to the manufacturer's recommendations.

### RNA extraction and quantitative real-time RT-PCR analysis

Total RNA was isolated from a minimum of 10^5^ iRBC-stimulated total PBMCs or MACS-sorted CD4^+^CD25^−^ and CD4^+^CD25^hi^ T cells using the RNeasy kit (Qiagen, Hilden, Germany) according to the manufacturer's recommendations. cDNA was synthesized using multiscribe reverse transcriptase and random hexamer primers in the presence of RNase inhibitor (Applied Biosystems, Foster City, CA) at 42°C for 15 min, followed by 5 min at 99°C. Foxp3, IL-10, TGFβ and 18SrRNA-specific primers and TaqMan probes were designed using Primer Express software (Applied Biosystems) and synthesized by Geneworks (primers) and Applied Biosystems (probes) (Table 1). Quantitative real-time PCR was performed in duplicate on an ABI PRISM 7900 (Applied Biosystems) as follows: 50°C for 2 min, 95°C for 10 min followed by 40 cycles of 95°C for 15 s and 60°C for 1 min. Results for target genes were normalized to 18SrRNA expression and expressed as fold changes between the cell populations of interest.

**Table 1:** Probe and primer sequences for quantitative real-time PCR analysis.

18SrRNA	Forward primer	5′-TCGAGGCCCTGTAATTGGAA-3′
	Reverse primer	5′-CCCTCCAATGGATCCTCGTT-3′
	Probe	5′- AGTCCACTTTAAATCCTT-3′
IL-10	Forward primer	5′-GGCGCTGTCATCGATTTCTT-3′
	Reverse primer	5′-TCTCTTGGAGCTTATTAAAGGCATTC-3′
	Probe	5′-CAAGAGCAAGGCCGTGGAGCAGG-3′
Foxp3	Forward primer	5′- GAGAAGCTGAGTGCCATGCA -3′
	Reverse primer	5′ – 5′ – GGAGCCCTTGTCGGATGAT -3′
	Probe	5′ – CCACCTGGCTGGGAAAATGGCAC -3′
TGFβ1	Forward primer	5′- CACCCGCGTGCTAATGG -3′
	Reverse primer	5′- ATGCTGTGTGTACTCTGCTTGAACT -3′
	Probe	5′- CCACAACGAAATCTA -3′

### Data presentation and statistical analysis

In most graphs depicted in this manuscript, we employed normalization onto control conditions for each donor, to be able to analyze changes in Foxp3^hi^ or Foxp3^int^ proportions (measured as percentage of CD4 T cells), surface marker expression levels (measured as median fluorescence intensity (MFI) or percent positive cells) or cytokine levels (measured in pg/ml) independent of variation of absolute values between different human donors. Normalized values are referred to as fold change compared to control conditions. To allow immediate identification, control conditions onto which other data in an experiment were normalized for each donor (value 1) are depicted as white bars, as opposed to black bars for all other conditions throughout. Statistical analysis was carried out using GraphPad Prism software v4 (San Diego, CA, USA). *P* values were determined by either Wilcoxon's signed rank test for absolute values, or two-tailed paired Student's *t*-test for normalized values. Three or more groups were compared by repeated-measures one-way ANOVA, followed by the linear trend, Dunnett's or Neuman-Keuls' multiple comparison post test. Correlation analysis was performed using Spearman rank correlation. A *P* value<0.05 was considered significant.
